# Systematic review of epidemiology, presentation, and management of Meckel's diverticulum in the 21st century

**DOI:** 10.1097/MD.0000000000012154

**Published:** 2018-08-21

**Authors:** Carl-Christian Hansen, Kjetil Søreide

**Affiliations:** a Medical Faculty, University of Bergen, Bergen; b Department of Gastrointestinal Surgery, Stavanger University Hospital, Stavanger; c Department of Clinical Medicine, University of Bergen, Bergen, Norway.

**Keywords:** ectopic tissue, epidemiology, Meckel's diverticulum, surgery, symptoms

## Abstract

**Background:**

The contemporary demographics and prevalence of Meckel's diverticulum, clinical presentation and management is not well described. Thus, this article aims to review the recent literature concerning Meckel's diverticulum.

**Methods:**

A systematic PubMed/Medline database search using the terms “Meckel” and “Meckel's” combined with “diverticulum.” English language articles published from January 1, 2000 to July 31, 2017 were considered. Studies reporting on the epidemiology of Meckel's diverticulum were included.

**Results:**

Of 857 articles meeting the initial search criteria, 92 articles were selected. Only 4 studies were prospective. The prevalence is reported between 0.3% and 2.9% in the general population. Meckels’ diverticulum is located 7 to 200 cm proximal to the ileocecal valve (mean 52.4 cm), it is 0.4 to 11.0 cm long (mean 3.05 cm), 0.3 to 7.0 cm in diameter (mean 1.58 cm), and presents with symptoms in 4% to 9% of patients. The male-to-female (M:F 1.5–4:1) gender distribution is reported up to 4 times more frequent in men. Symptomatic patients are usually young. Of the pediatric symptomatic patients, 46.7% have obstruction, 25.3% have hemorrhage, and 19.5% have inflammation as presenting symptom. Corresponding values for adults are 35.6%, 27.3%, and 29.4%. Ectopic gastric tissue is present in 24.2% to 71.0% of symptomatic Meckel's diverticulum, is associated with hemorrhage and is the most common form of ectopic tissue, followed by ectopic pancreatic tissue present in 0% to 12.0%.

**Conclusion:**

The epidemiological patterns and clinical presentation appears stable in the 21st century. A symptomatic Meckel's diverticulum is managed by resection. The issue of prophylactic in incidental Meckel's diverticulum resection remains controversial.

## 1 Introduction

A Meckel's diverticulum is a relatively common congenital diverticulum on the ileum resulting from incomplete atrophy of the vitelline duct in the embryo.^[1,2]^ The name is derived from the German anatomist Johann Friedrich Meckel who described this entity in the early nineteenth century.^[3]^ A Meckel's is an obscure feature of human anatomy sometimes acknowledged during abdominal surgery. Even though the majority of Meckel's never become symptomatic, their potential to present with severe complications such as bleeding or perforation has nevertheless caused much debate regarding whether a silent Meckel's should be preemptively resected when incidentally discovered during surgery. To the best of our knowledge, this question has not yet been settled. In addition, with advances being made in medicine, like the advent of laparoscopic surgery and improved imaging studies like computed tomography (CT), the epidemiology of Meckel's needs to be reassessed.

Thus, the purpose of this article is to review the recent literature in the 21st century concerning Meckel's epidemiology, patterns of presentation and management. The aim is to investigate the prevalence and incidence of Meckel's, the size and location, the age and gender distribution of the patients, clinical presentation, and the presence of ectopic tissue. We are also interested to see if practice to perform the prophylactic resection of incidentally detected Meckel's has changed.

## 2 Materials and methods

### 2.1 Ethics

No ethical approval was required for this study as this was a literature review.

### 2.2 Search strategy

The PubMed online search engine was searched using the words “Meckel” or Meckel's” combined with “diverticulum.” The search was concluded 08.01.2018, and was limited to English language articles reporting on human subjects and published between 01.01.2000 and 30.06.2017. We screened for relevant articles, and selected articles for further reading based on the title and abstract. We selected and read in full articles reporting on patient series or hospital database searches for patients with Meckel's, while excluding case studies and reports on patient series with <4 patients. We considered articles relevant if they reported on the epidemiology of Meckel's, such as prevalence, incidence, presentation, sex, and age; on properties of the Meckel's itself, such as histology, location, and morphology; or on the management of Meckel's. For location and morphology of the Meckel's, we summarized in a quantitative way the reported mean values in the literature and derived a weighted mean of the means. For other results, we compiled several tables, in which the results from the largest patient series were included. We consulted the PRISMA statement for systematic reviews during the design, search, and writing of this article.^[4]^

## 3 Results

**Figure 1:**
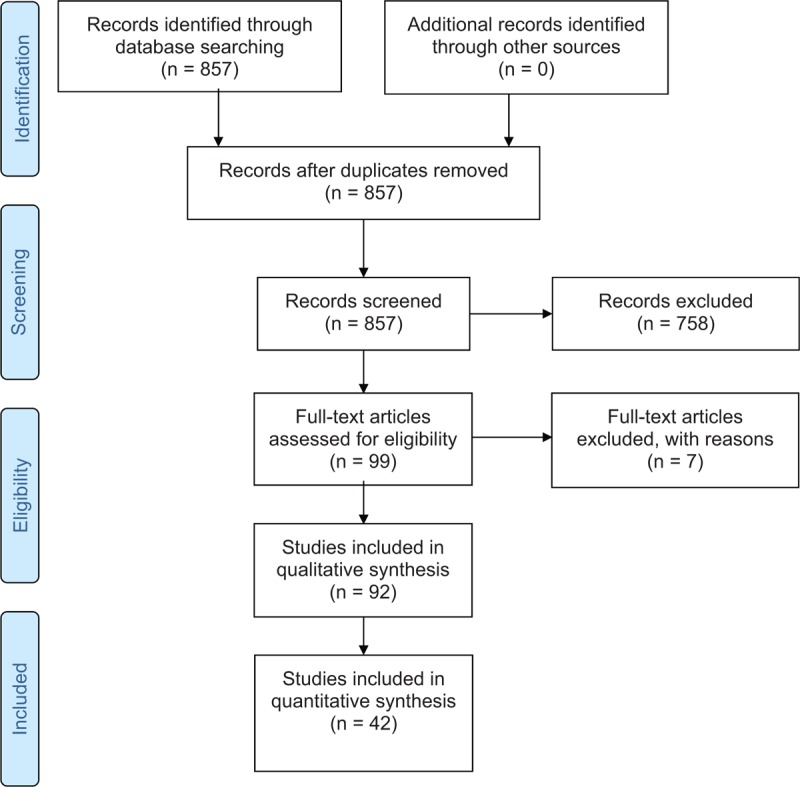
PRISMA flow chart for the literature search.

Of the original 857 articles, we selected 99 articles for further reading based on a screening of the title and abstract. After excluding seven articles following a full text screen, we included 92 articles in the study, as presented in the PRISMA flow-chart (Fig. 1).

### 3.1 Epidemiology

The reported prevalence of Meckel's is between 0.3% and 2.9% of the general population (Table 1) based on 8 studies.^[5–12]^ Zani et al^[8]^ published a review of earlier autopsy-studies, arriving at a prevalence of 1.2%. Other studies have arrived at similar numbers; those studies are for the most part single-centre retrospective studies where the prevalence was determined as the share of patients found to have Meckel's during appendectomies^[5,7,9,11,12]^ or abdominal surgery.^[10]^ In one study, the prevalence was determined as the proportion of patients with Meckel's among patients with Crohn's disease, reasoning that Crohn's entails thorough intestinal investigation which would allow for accuracy in determining presence of a Meckel.^[6]^

**Table 1:**
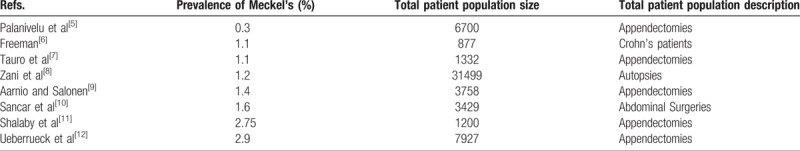
The prevalence of Meckel's diverticulum.

### 3.2 Symptomatic vs silent disease

In the largest patient series (each containing >100 patients), the proportion of symptomatic Meckel's is 9.0% to 71.1% of all resected specimens (see Table 2,^[12–18]^). These numbers are the result of retrospective reviews,^[12,13,15,17,18]^ or derived from databases containing patient information.^[14,16]^ Ueberrueck et al^[12]^ reported 9% symptomatic Meckel's from a large patient series (233 patients) that included many silent Meckel's. This was due to their deliberate search for silent Meckel's during appendectomies.^[12]^ Zani et al^[8]^ combining the prevalence from the autopsy studies they reviewed with the reported number of hospital admissions due to symptomatic Meckel arrived at an estimated 4.2% lifetime incidence of symptomatic Meckel's. In comparison, the lifetime incidence risk for appendicitis is reported at 7% to 8%.^[19]^

**Table 2:**
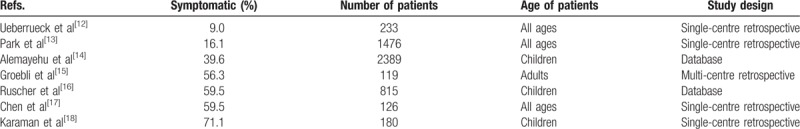
Proportion of symptomatic Meckel's diverticula.

Most patients with symptomatic or resected Meckel's are male. The largest retrospective patient series (each containing >100 patients) report a male to female gender ratio of 1.5:1 to 4:1 (see Table 3,^[12–18,20,21]^). The same is true for the database queries performed by Alemayehu et al^[14]^ and Ruscher et al.^[16]^

**Table 3:**
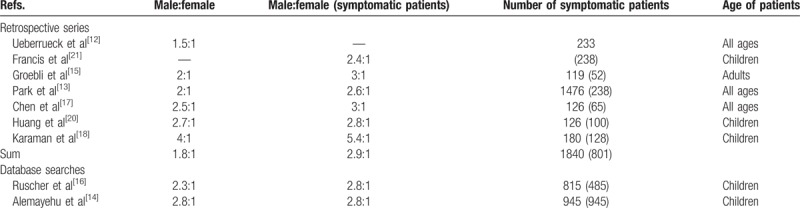
The gender distribution of Meckel's diverticulum.

A symptomatic Meckel's can present at all ages,^[22]^ but it is a condition predominantly presenting in children. This is shown by Alemayehu et al^[14]^ and Ruscher et al,^[16]^ who found that more than half of all children with Meckel's who required surgery were <5 years. The retrospective series that contain patients of all ages and stratify them by age agree that the prevalence of symptomatic disease decreases with age,^[9,12,13,23–25]^ with several of them finding that more than half of all symptomatic patients were younger than 10 years.^[23–25]^

### 3.3 Localization of Meckel's

Meckel's, when present, is located 7 to 200 cm from the ileocecal valve on the antimesenteric margin of the ileum. A weighted mean of the reported mean distances places the Meckel's at 52.4 cm from the ileocecal valve (combined number of patients = 423). It is 0.4 to 11 cm long with a diameter of 0.3 to 7 cm, with a weighted mean length of 3.05 cm (combined number of patients = 595) and diameter of 1.58 cm (combined number of patients = 581).^[9,20,23–34]^

### 3.4 Cause of symptomatic Meckel's diverticulum

The most common etiologies of symptomatic Meckel's are intestinal obstruction, gastrointestinal (GI) hemorrhage, and inflammation of the Meckel's with or without perforation.

Obstruction refers to instances in which the Meckel's is the cause of intestinal obstruction, for instance by intussusception or invagination of the Meckel's into the lumen of the small intestine. Volvulus of the small intestine around the diverticular axis is another possible mechanism. GI-hemorrhage refers to painless bleeding per rectum and is often the result of acid produced from a patch of ectopic gastric mucosa in the Meckel's damaging the intestinal lumen, leading to a bleeding ulcer. Inflammation refers to either inflammation of the Meckel's itself or perforation of the diverticular walls resulting in peritonitis.

Combining the largest pediatric patient series (each series containing >50 symptomatic patients), 46.7% of children with symptomatic Meckel's present with obstruction, 25.3% present with GI-hemorrhage, and 19.5% present with inflammation. Searching in the Paediatric Hospital Information System Database for children with symptomatic Meckel's, Alemayehu et al^[14]^ found 60.1% of children presenting with obstruction, 35.6% presenting with GI-hemorrhage, and 8.4% presenting with inflammation. In the largest adult series (each series containing >20 symptomatic patients), 35.6% of adults present with obstruction, 27.3% present with GI-hemorrhage, and 29.4% present with inflammation (see also Table 4,^[13,15,17,18,20,28,35–41]^). Added together, obstruction, hemorrhage, and inflammation account for 69.5% to 100% of symptomatic patients in each of the largest retrospective patient series. Rarer forms of symptomatic Meckel's, including umbilical abnormalities involving the vitelline duct,^[39]^ parasite-infections involving the Meckel's,^[42]^ Meckelian cancers,^[43]^ as well as uncertain cases,^[28]^ account for the remainder.

**Table 4:**
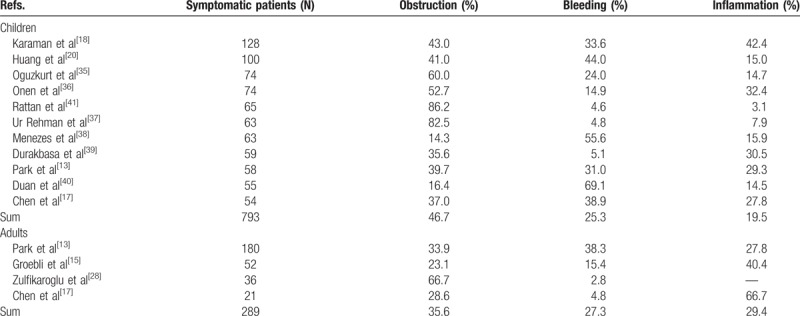
Etiology of symptomatic Meckel's diverticulum in children and adults.

### 3.5 Ectopic tissue

The intestinal mucosa lining the walls of the ileum also line the walls of the Meckel's, but frequently the Meckel's contains ectopic tissue. Present in 4.6% to 71.0% of symptomatic Meckel's, gastric tissue is the most common, followed by pancreatic tissue present in 0% to 12.0%, see Table 5.^[13,17,18,20,21,23,24,26,28,35,38,39,41]^ Articles are included in Table 5 if they differentiated between symptomatic and silent Meckel's and number of patients were >50.

**Table 5:**
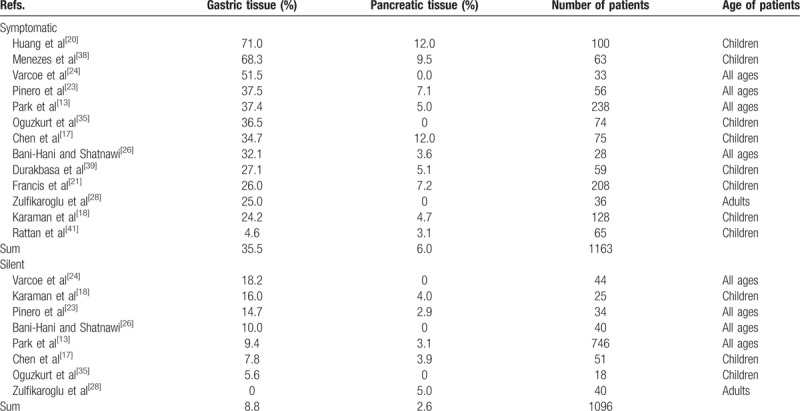
Ectopic tissue in symptomatic and silent Meckel's diverticulum.

Ectopic tissue is occasionally present in silent Meckel's, but to a lesser degree: 0% to 18.2% contain ectopic gastric tissue while 0% to 5.0% contain ectopic pancreatic tissue. Together, ectopic gastric and pancreatic tissue account for 97.0% of all ectopic tissues in the studies cited in Table 5. Rarer forms include ectopic duodenal and colonic tissue.^[13]^

The presence of ectopic tissue is associated with symptomatic Meckel's in general,^[13,25,26,44]^ and with GI-hemorrhage in particular.^[17,20,23,24,28,34,35,45,46]^ Of the patient series in Table 5 in which the authors disclosed the number of patients with hemorrhage and ectopic gastric tissue, 77% (63–100% in each series, combined number of patients = 186) of patients with hemorrhage had ectopic gastric tissue.^[13,17,20,24,35,39,41]^ In a meta-analysis, Burjonrappa and Khaing^[44]^ confirmed that the presence of ectopic tissue is the most significant factor determining the need for surgical intervention in patients with Meckel's. In their own patient series of 22 children, they also found that symptomatic pediatric patients without ectopic tissue were younger than symptomatic pediatric patients with ectopic tissue.^[44]^

### 3.6 Determination of ectopic tissue

There is no reliable way to tell whether any given silent Meckel's contains ectopic tissue just by looking at it. Palpable thickening of the Meckel's was thought to indicate the presence of ectopic tissue, but none of the patient series that investigated this was able to demonstrate any association.^[13,18,33]^ There is, however, some evidence supporting the notion that the height-to-diameter ratio influences the distribution of the ectopic tissue within the Meckel's. Two smaller patient series (Meckel's measured = 30^[11]^ and 8^[47]^) found that when the height-to-diameter ratio was greater than 1.6, the ectopic tissue was located exclusively in the tip, while for ratios less than 1.6 the ectopic tissue could also include the base of the Meckel's. In a slightly larger patient series, the same phenomenon was observed for a ratio of 2 (Meckel's measured = 25^[24]^) One possible explanation for this is the pluripotent cell theory of ectopic tissue origin, which posits that ectopic tissue originates from pluripotent cells in the embryonic yolk sac, which communicates with the vitelline duct.^[47]^

### 3.7 Age and relation to symptoms

Age seem to correspond with certain presentation of specific complications. While obstruction and GI-hemorrhage are both common presentations in pediatric patients,^[17,20,23,36]^ patients with obstruction seem to be younger.^[13,34]^ This was not clear in all patient series; according to Park et al,^[13]^ patients younger than 11 years tended to present with obstruction, and patients younger than 4 especially, but this only became apparent after defining pediatric patients as younger than 11 years. Had they not done so, they would have found that bleeding was the most common presentation in children. Inflammation and Littre hernia (hernia involving the bowel segment bearing Meckel's) are more common in adults,^[17,23]^ while cancers develop in older patients.^[43]^

Obstruction, GI-hemorrhage, and inflammation are all relatively common in both adult and pediatric patients (Table 3). Therefore, the importance of age and the likelihood of the presenting symptoms and complication a Meckel's may present with is important to keep in mind. Only certain, rare forms of symptomatic Meckel's are very restrictive concerning the age groups in which they appear. While Meckel's is the result of incomplete atrophy of the vitelline duct, a patent vitelline duct may communicate between the small intestine and the umbilicus and lead to umbilical discharge. This condition and others, like umbilical hernias involving Meckel's, are congenital and diagnosed quickly after birth.^[39]^ Meckelian cancers, which are cancers originating from or involving the Meckel's, have a mean age at diagnosis of 60 years.^[43]^

### 3.8 Diagnosis

As stated above, symptomatic Meckel's can present as mechanical obstruction of the small bowel, either due to intussusception or in some other way. It can also present as painless bleeding per rectum, or with signs of inflamed Meckel's or peritonitis. Common symptoms are fever, vomiting, abdominal pain, and bloody stools.^[20]^ These symptoms, and the pathological processes that cause them, are not unique to Meckel's. For instance, an inflamed or perforated Meckel's may be mistaken for an inflamed appendix,^[5]^ a much more common condition. Therefore, Meckel's represents a diagnostic challenge and are often incidentally found during work up for symptoms though to be of another cause.

Meckel's can be diagnosed by using imaging modalities like ultrasound, X-ray, angiography, CT, and magnetic resonance imaging, but the sensitivity and specificity is low.^[9,15,24,48]^ They are not without value, though, as they can show small-bowel obstruction and intussusception and lead to correct surgical interventions,^[20]^ and finding a normal appendix on such tests can encourage the radiologist to consider differential diagnoses like symptomatic Meckel's.^[5,49]^ Angiography may identify the source of GI-hemorrhage, and the vitelline artery branching off the superior mesenteric artery, when present, is pathognomonic for Meckel's.^[48]^ When observed on ultrasound and computer tomography, the Meckel's takes the shape of a cyst or blind pouch diverging from the ileum.^[49,50]^ It can be difficult to discern the Meckel's from the adjacent loops in the small intestine, but sometimes an attached band tethering the Meckel's to the umbilicus or mesentery offer additional aid in finding the right diagnosis.^[49,50]^ The amount of peritoneal fat, separating the bowel loops from each other, may increase the chances of detection on CT images, but in the end, in order to find Meckel's on CT images, one needs to actively search for it.^[51]^

Nuclear scans with Tc-99m pertechnetate may visualize the Meckel's, taking advantage of the way the tracer accumulates in certain tissues like ectopic gastric tissue, which is sometimes found in the Meckel's. Several of the articles reviewed focused on the diagnostic value of this test, and of 562 scans, 83 were positive while 479 were negative.^[11,46,52–57]^ Sixty-nine were true positive, 14 were false positive, and while 8 were found to be false negative, 471 were assumed to be true negative. This gives a sensitivity of 89.6% and a specificity of 97.1% (Table 6). When positive, the test should display focal uptake simultaneous with the gastric tissue in the stomach.^[55]^ Several factors influence the result: true positive results hinge on the presence of functional ectopic gastric mucosa in the Meckel's, as the test is really a test for ectopic gastric tissue, which has to be present in sufficient amounts. Bleeding may cause extravasation of tracer, potentially causing both false positives and negatives. The Meckel's may also lie hidden behind another structure that accumulates tracer, such as the stomach, the kidneys, or the bladder. Premedication with certain drugs has been introduced to increase the diagnostic value of the test. Examples are H2-antagonists like cimetidine or ranitidine to prevent secretion of tracer by the gastric cells and stimulate accumulation.^[45]^ Repeat scans when results are inconclusive or clinical suspicion is high is also a viable option.^[58]^ Restricting use of the test to certain indications, such as anemic patients with GI-hemorrhage, is also important to ensure high sensitivity and specificity.^[57]^

**Table 6:**
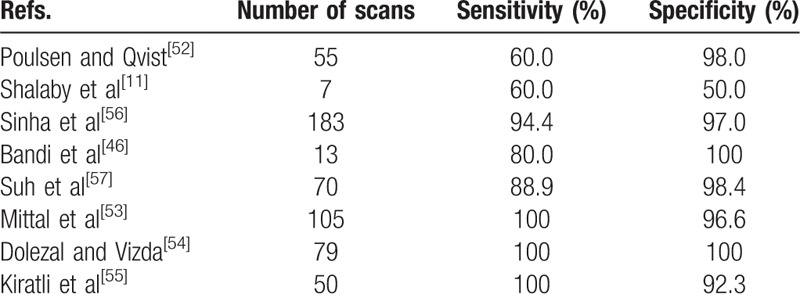
Sensitivity and specificity of Tc-99m pertechnetate nuclear scans.

Direct observation of the Meckel's will yield the correct diagnosis. This can be done surgically, either by laparoscopy^[59]^ or laparotomy,^[31]^ or with endoscopy of the small intestine.^[60]^ Double-balloon endoscopy is a technique that allows the endoscope to travel further into the ileum until the Meckel's is found.^[60]^ Capsule endoscopy is a different technique where a swallowed camera records the bowels while they propel it forward.^[34]^ A downside to the capsule endoscopy is the lack of control, as it may move past the opening of the Meckel's before it is able to record it, or the camera may be facing the wrong direction when passing the mouth of the Meckel's. In a study by He et al^[61]^ comparing the 2 advanced endoscopy techniques, double-balloon endoscopy was able to observe 64 of 74 possible Meckel's. Out of 26 patients who underwent both techniques, 20 of 22 Meckel's detected on double-balloon endoscopy went undetected on capsule endoscopy. The 10 Meckel's that went undetected by double-balloon endoscopy were subsequently found on surgery.^[61]^

### 3.9 Management

The treatment for symptomatic Meckel's is resection, either laparoscopically or by way of open surgery, with or without a wedge or segment of the adjacent intestine.^[20]^ Several laparoscopic or laparoscopy-assisted techniques allow for the resection of Meckel's, by way of 1 trocar or 3,^[62]^ and by resection intraperitoneally or by exteriorization of the intestinal segment bearing the Meckel's through an abdominal incision.^[29,62]^ Laparoscopic or laparoscopy assisted resection of Meckel's is described as safe and effective.^[29,62]^

### 3.10 Complications after surgery

Complications following resection of Meckel's can occur. In a systematic review by Zani et al, the postoperative morbidity was 5.3%, with wound infections being the most common complication. Together with postoperative ileus, it accounted for 66% of all postoperative complications, and the morbidity was found to be higher than for Meckel's left in situ.^[8]^ Among the patient series, a few compared resection of symptomatic Meckel's to resection of silent Meckel's and concluded that there are no discernible differences in the rates of morbidity and mortality.^[13,25,28]^ Ueberrueck et al^[12]^ even found a lower rate of morbidity for Meckel's resections than for appendectomies, and no difference between resection and nonresection of the Meckel's. The findings are not without significant caveats; since prophylactic resections are performed during surgery for reasons unrelated to the Meckel's, it is not a simple matter of assigning responsibility for postoperative deaths and complications, except in obvious circumstances.^[13]^ Furthermore, many surgeons take a differentiated approach to prophylactic resections. In the study by Ueberrueck et al,^[12]^ a perforated or gangrenous appendix was considered contraindication for prophylactic resection of Meckel's, thereby selecting gangrenous and perforated appendices for the nonresection group and less severe cases of acute appendicitis for the resection group.

## 4 Discussion

The present study is a systematic literature overview of all cohort series on Meckel's diverticulum published after year 2000. The collated information represents the current state-of-the art overview of epidemiology, presentation, and management in the 21st century. As also found in older studies, nearly all patient series reviewed for this article had a retrospective design. Only 4 articles included prospective studies. This is not ideal, but understandable due to the characteristics of the Meckel's. It is present in a minority of the population and expresses itself in a mere minority of the minority. For the most part, it remains silent and well hidden. The prevalence of Meckel's has been reported at 0.3% to 2.9%, with results from retrospective studies agreeing with the prevalence derived from a systematic review of autopsy-studies.^[8]^ Retrospective studies can be unreliable when examining the incidence of symptomatic Meckel's, since many silent Meckel's go undetected. However, in a retrospective study of 233 patients with Meckel's,^[12]^ a deliberate search for Meckel's was made during all appendectomies. This resulted in the lowest proportion of symptomatic Meckel's (9%) in a retrospective study, and likely provides for one of the better estimates. While some of the silent Meckel's removed could have become symptomatic later had they remained in situ, they weigh up for the few silent Meckel's that may have gone undetected even with the surgeon deliberately searching for them. By a different approach, Zani et al^[8]^ calculated a 4.2% life-time risk of developing complications, which happens to agree very nicely with the earlier 6.4% estimate by Cullen et al^[2]^ and 4.2% estimate by Soltero and Bill.^[63]^ Together, these studies suggest the life-time risk for symptomatic Meckel's to be at 4.2% to 9.0%.

The studies agree there are more men than women presenting with symptomatic Meckel's, and that while symptomatic Meckel's can occur at any age, it is more frequently associated with younger age. Studies agree that the 3 most common presentations of symptomatic Meckel's are caused by obstruction, GI-hemorrhage, and inflammation with or without perforation, and that ectopic gastric tissue is associated with symptomatic Meckel's in general and with GI-hemorrhage particularly. There is also agreement that ectopic gastric tissue is the most common form of ectopic tissue found in the Meckel's, followed by ectopic pancreatic tissue.

Interesting explanations for some of these observations have been proposed. In a small study on the nervous structure of Meckel's in children,^[64]^ it is suggested that the nerve fiber density of the diverticular walls is a factor. They found a higher nerve fiber density in the walls of the Meckel's lined with intestinal mucosa compared to areas lined with ectopic gastric mucosa and the walls of the ileum. Proposing that higher nerve fiber density leads to more intense local peristalsis that may cause intussusception of the Meckel's, and that the nerve fiber density decreases with age, a neat explanation emerges for why symptomatic Meckel's presents more often in young patients. In a retrospective study of 47 children,^[34]^ the authors propose that acid production in ectopic gastric mucosa increases with age, which together with the above-mentioned suggestion concerning nerve fiber density^[64]^ could help explain why children presenting with obstruction caused by Meckel's are sometimes found to be younger than children presenting with hemorrhage caused by Meckel's. Lastly, in a retrospective study on 100 children with Meckel's,^[20]^ the authors propose that higher acid-production in males help account for the gender-distribution, citing increased risk for peptic ulcers in men and their own series of patients with Meckel's, in which the male preponderance was especially pronounced in nonobstruction symptomatic patients.

A mnemonic describing the characteristics of Meckel's, the so-called “rule of 2's”, states that general characteristics of the Meckel's can be summarized using 2's: it is found in 2% of the population, 2 feet (about 61 cm) from the ileocecal valve, and is 2 in. (about 5 cm) long. There are 2 common forms of ectopic tissue, and the most common age at presentation is 2 years.^[1]^ Though the “rule of 2's” was not confirmed, the literature does give some credit to the approximation as a rule of thumb. According to the rule, a 2% prevalence is expected, which is in agreement with the literature. According to the rule, one would expect to find the Meckel's 2 feet (61 cm) from the ileocecal valve, which is close to the weighted mean of 52.4 cm. As for the length of the Meckel's, the rule says 2 inches, but we found a weighted mean of 3.05 cm, which is closer to 1 in. (2.54 cm). In accordance with the rule, there are 2 common types of ectopic tissue, namely ectopic gastric and pancreatic tissue. Lastly, with median ages of 4 and 5 years as reported in 2 database queries,^[14,16]^ we can infer that 2 years is a rather common age for presenting with symptomatic Meckel's. The values found may not line up exactly according to the rule on every item, but the rule was never far from the values found in or derived from the literature. It is worth remembering that “the rule” is a mnemonic that, while based on empirical evidence, allows certain compromise for the sake of simplicity.

The real controversy surrounding Meckel's concerns the option of treating silent Meckel's with prophylactic resection when discovered during surgery. Some advise against prophylactic resection, arguing that the morbidity is too high and that the reward is too low. Zani et al^[8]^ takes this position after conducting a systematic review and finding a 5.3% risk of postoperative complications after prophylactic resection and a 1.3% risk of developing symptoms after leaving it in situ. They also found no long-term complications associated with leaving the Meckel's in situ when reviewing articles that reported follow-up on patients with silent Meckel's left in situ, and estimated that more than 750 silent Meckel's would have to be resected in order to save one life.^[8]^ Soltero and Bill^[63]^ reached a similar conclusion, arguing against prophylactic resection after estimating that more than 800 prophylactic resections would have to be made in order to save one life. This view is not held by Cullen et al,^[2]^ who found that the risk of developing symptomatic Meckel's did not decrease with age, and who held that prophylactic resection is recommended except in the face of contraindications like generalized peritonitis or other conditions that make resection more hazardous.

The retrospective studies are also not in harmony with each other on this subject. Many authors base their recommendations on their own experiences and patient series, and while practical experience does matter, different experiences and perspectives may lead to contradicting recommendations. In a smaller patient series of seven, the authors enthusiastically supports prophylactic resection, arguing that if their patients had had a search for Meckel's and prophylactic resection when they underwent appendectomy, they would have completely avoided developing symptomatic Meckel's later.^[65]^ Another set of authors, reporting on a patient series of 50 patients in which 40% of patients developed potentially life-threatening symptomatic Meckel's, also favor prophylactic resection.^[33]^ A third set of authors, after encountering potentially life threatening postoperative complications in their patient series of 47 patients, advocate against prophylactic resection.^[66]^ Lastly, in a population-based epidemiological study on Meckelian cancers, the authors advocate for prophylactic resection after finding the Meckel's has a 70-fold higher risk of cancer development than any other site in the ileum, and that the mean age at diagnosis was 60 years.^[43]^

Other authors choose a differentiated approach, advocating for prophylactic resection upon meeting certain criteria that increase the likelihood of the silent Meckel's becoming symptomatic. The largest of the retrospective patient series from 2000 to 2017 identified 4 such criteria: male sex, younger than 50 years, greater diverticular length than 2 cm, and the presence of ectopic tissue.^[13]^ When meeting up to all of these criteria, 17%, 25%, 42%, and 70% of Meckel's were symptomatic.

With the different perspectives in mind, a differentiated approach seems the most appropriate. In pediatric patients, one should resect silent Meckel's discovered incidentally during surgery. In adult patients, one should resect incidentally discovered Meckel's that have traits associated with complications, such as length greater than 2 cm. In elderly patients, one should leave the silent Meckel's in situ. Additionally, one should keep the importance and seriousness of the ongoing surgery in mind before deciding to remove or leave behind a silent Meckel's. A silent Meckel's may not take precedence over immediate matters of life and death.

When choosing between the different procedures for the resection of a Meckel's, diverticulectomy and segmental resection of the ileum should be preferred in broad-based Meckel's to avoid restricting the intestinal lumen, as well as in bleeding Meckel's to ensure full resection of any ectopic gastric tissue and intestinal ulcer. Peptic ulcers resulting from ectopic gastric acid production are often located in the ileum rather than the MD itself, due to peristaltic activity in the Meckel's and the resistance of the ectopic gastric tissue to the acid it produces.^[24,47,64,67]^ For long and thin Meckel's without hemorrhage, a simple resection should suffice, as any ectopic tissue within is likely to be confined to the tip.^[11,24,47]^

## 5 Conclusion

The general properties of Meckel's are stable and well described in the recent literature, which for the most part consists of retrospective studies. Symptomatic Meckel's is managed by surgical resection, but the issue of prophylactic resection remains controversial and unresolved.

## Author contributions

**Conceptualization:** Kjetil Soreide.

**Data curation:** Carl-Christian Hansen, Kjetil Soreide.

**Methodology:** Carl-Christian Hansen, Kjetil Soreide.

**Resources:** Carl-Christian Hansen.

**Software:** Carl-Christian Hansen, Kjetil Soreide.

**Writing – original draft:** Carl-Christian Hansen, Kjetil Soreide.

**Writing – review & editing:** Carl-Christian Hansen, Kjetil Soreide.
